# Is 3-Carboxy-4-methyl-5-propyl-2-furanpropionate (CMPF) a Clinically Relevant Uremic Toxin in Haemodialysis Patients?

**DOI:** 10.3390/toxins10050205

**Published:** 2018-05-18

**Authors:** Mathilde Luce, Anais Bouchara, Myriam Pastural, Samuel Granjon, Jean Christophe Szelag, Maurice Laville, Walid Arkouche, Denis Fouque, Christophe O. Soulage, Laetitia Koppe

**Affiliations:** 1 Department of Nephrology, Hospices Civils de Lyon, Centre Hospitalier Lyon-Sud, F-69495 Pierre-Benite, France; mathilde.luce1@gmail.com (Mathilde L.); anais.bouchara@chu-lyon.fr (A.B.); maurice.laville@chu-lyon.fr (Maurice L.); denis.fouque@univ-lyon1.fr (D.F.); 2 University of Lyon, CarMeN lab, INSERM U1060, INRA U1397, INSA de Lyon, Université Claude Bernard Lyon 1, F-69621 Villeurbanne, France; christophe.soulage@insa-lyon.fr; 3 Association Pour l’Utilisation du Rein Artificiel dans la Region Lyonnaise (AURAL), F-69008 Lyon, France; myriam.pastural@auralyon.com (M.P.); jcszelag@auralyon.com (J.C.S.); warkouche@aurar.fr (W.A.); 4 Laboratoire d’Analyse Medicale Cerballiance Rhone alpes, F-69008 Lyon, France; samuel.granjon@cerballiance.fr

**Keywords:** CMPF, Uremic toxins, CKD, haemodialysis, protein energy wasting, nutrition

## Abstract

3-Carboxy-4-methyl-5-propyl-2-furanpropionate (CMPF) is a metabolite of furan fatty acid and a marker of fish oil intake. CMPF is described as a protein-bound uremic toxin and interacts with free oxygen radicals, which can induce cell damages. However, the clinical consequences of CMPF accumulation in haemodialysis patients remain poorly documented. The aims of this study are to investigate potential association between CMPF levels and (i) biochemical and nutritional parameters; (ii) cardiovascular events and (iii) mortality. Two hundred and fifty-two patients undergoing maintenance haemodialysis were included. Routine clinical biochemistry tests and assay for CMPF by HPLC technique were performed at the inclusion. Body composition parameters were measured using a bioimpedance spectroscopy method. The enrolled patients were prospectively monitored for cardiovascular events and mortality. CMPF level was positively correlated with nutritional parameters and lean mass and is significantly higher in patients without protein-energy wasting. However, the multivariate linear regression analysis indicated that CMPF level was not independently associated with albumin, prealbumin, creatinemia and body mass index. Elevated serum CMPF was not associated with mortality and cardiovascular morbidity. Our results indicate that CMPF is not a relevant uremic toxin in haemodialysis and in contrast could be a marker of healthy diet and omega 3 intakes.

## 1. Introduction

Chronic kidney disease (CKD) is characterized by accumulation of uremic toxins especially, some protein-bound uremic toxins as p-cresyl sulfate and indoxyl sulfate. The clinical manifestations of these uremic toxins are rather nonspecific and may include neurologic disorders, protein energy wasting (PEW), cardiovascular (CV) diseases, progression of CKD and mortality [1,2,3].

3-Carboxy-4-methyl-5-propyl-2-furanpropionate (CMPF) is a one of the major endogenous metabolites of furan fatty acids (Furan FAs). Furan FAs are incorporated into phospholipids and cholesterol esters and are catabolized into dibasic urofuran acids excreted in the urine. CMPF showed high protein-binding ratios (more than 95%) and it is poorly removed by haemodialysis (HD). Consequently, blood CMPF levels are elevated in CKD [4]. However, the source of elevated circulating CMPF levels is unknown. The richest sources of Furan FAs in food are fish and fish oils and, consumption of fish is associated with increased plasma CMPF in healthy patients. Green vegetables, champignons, algae, soy beans and wheat germ oil contain lowest Furan FAs but plasma CMPF did not correlate with intakes of these foods [5]. 

The metabolic consequences of CMPF accumulation are unclear and still under debate [6,7]. In the one hand, seafood based alimentation is generally considered as a healthy food and even appears to protect from type 2 diabetes or inflammation in observational studies [8], though this is not seen in all studies [9]. Therefore, CMPF has been suggested as a specific biomarker for fatty fish intake and healthy diet intervention [10,11]. In the other hand, CMPF seems to be a predictive biomarker in metabolic diseases. It was demonstrated that elevated CMPF is associated with development of gestational diabetes mellitus, metabolic syndrome or type 2 diabetes. Indeed, CMPF could directly promote β cells dysfunction, through mitochondrial dysfunction and oxidative stress [12]. However, other clinical studies found no deleterious impact of glucose metabolism resulting from CMPF accumulation [11,13].

In CKD, CMPF is described as a deleterious uremic toxin because CMPF directly interacts with free oxygen radicals, which can induce cell damages [14]. Therefore, in experimental studies, CMPF has been reported to inhibit erythropoiesis [15], contributes to the development of thyroid abnormalities [16], impairs neurological function [17] and leads to renal cellular damage [14]. Given its role in oxidative stress [18], CMPF is associated as an uremic retention toxins with CV relevance [19]. The uncertainties concerning CMPF toxicity also results from the wide range of values reported for plasma CMPF concentration and difficulties to interpret and compare studies. Surprisingly, the clinical consequences of CMPF accumulation in HD patients have up to now never been explored.

In order to assess if CMPF could be clinically involved in end-stage renal disease adverse outcomes, we investigated the correlation between plasmatic CMPF levels and (i) biochemical; nutritional parameters and body composition (ii) CV events and (iii) mortality in a large cohort of 238 maintenance HD patients.

## 2. Results

### 2.1. Participant Characteristics

**Figure 1:**
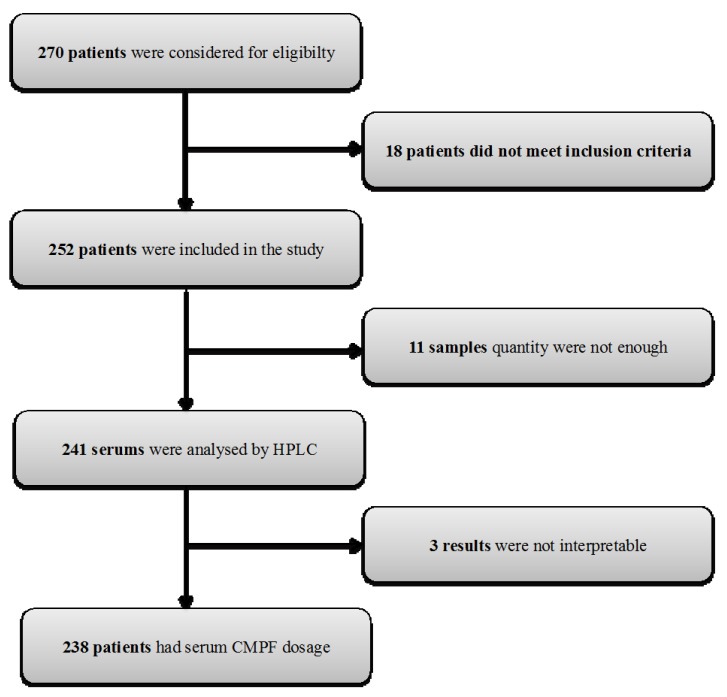
Flow chart of patients’ inclusion and CMPF procedure.

252 patients were included in the study. 11 patients were excluded because serum volume was too low to allow CMPF assay. For 3 patients, HPLC analysis procedure was uninterpretable. 238 patients had a complete serum CMPF dosage (Figure 1).

270 patients were considered for inclusion and 252 patients were included. Eleven samples could not be quantified because of missing serum. 238 patients (i.e., 94%) had a complete CMPF dosage by HPLC coupled to UV detection.

The main characteristics of the patients are detailed in Table 1. The median level of serum CMPF was 2.55 [1.00–5.2] mg/L. Briefly, the mean duration of follow-up was 937 ± 518 days. Median age was 64 [47.2–75.6] years old and dialysis vintage was 2.2 [1.1–21.9] years. 62% of the patients were males (*n* = 148). 33% of the patients had a medical history of CV events (*n* = 79) and 89% of the patients suffered hypertension (*n* = 212). 68 patients had type 2 diabetes (29%) and 131 patients had dyslipidemia (55%). The single pool Kt/V (spKt/V) was upper 1.4 per sessions: 1.7 [1.5–1.9] as recommended by the KDOQI guidelines [20].

### 2.2. CMPF Is a Marker of Nutritional Status and Body Composition

Patients with a serum CMPF level greater than or equal to the median value presented a higher creatinine index (CI), pre-dialysis creatinine serum levels (Cr_pre_), urea, albumin and prealbumin and a lower LDL cholesterol than those below the median value (Table S1). Univariate associations between CMPF and other variables in all patients are shown in Table 2. CMPF levels were positively related to dialysis vintage (r_s_ = 0.16, *p* = 0.01), albumin (r_s_ = 0.20, *p* = 0.003), prealbumin (r_s_ = 0.16, *p* = 0.02), Cr_pre_ (r_s_ =0.18, *p* = 0.004), CI (r_s_ = 0.16, *p* = 0.01) and body mass index (BMI) (r_s_ = 0.16, *p* = 0.01). There was no significant correlation with nPCR (r_s_ = −0.01, *p* = 0.89). In order to identify clinical biochemical parameters that might be independently associated with elevated CMPF levels in our population, we performed a multiple regression analysis. As presented in Table 3, Cr_pre_, BMI, albumin, prealbumin were not independently associated with CMPF plasma concentration.

**Figure 2:**
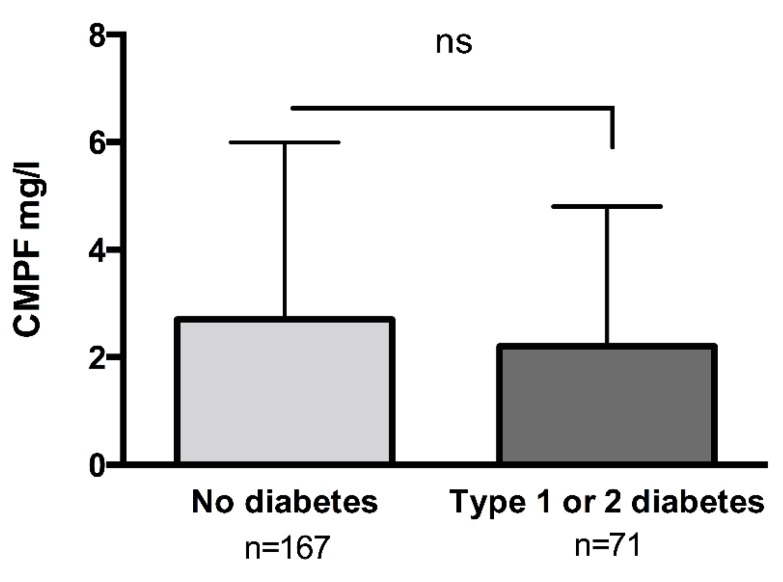
Plasma CMPF concentration does not depend on diabetic status in haemodialysis patients. Patients were divided into two groups: no history of diabetes (*n* = 167) or type 1 or 2 diabetes (*n* = 71). Data were analysed with Mann Whitney U test. Data are expressed as median [IQR]. Differences were considered significant at the *p* < 0.05 level. Abbreviation: ns, non-significant.

There was no association between CMPF and metabolic parameters such as glycaemia (r = −0.08, *p* = 0.22), HbA1C (r_s_ = −0.01, *p* = 0.87), or triglycerides (r_s_ = 0.08, *p* = 0.33) (Table 2). CMPF levels were not higher in patients with type 1 and 2 diabetes (2.7 mg/L [1.0–6.0] versus 2.2 mg/L [0.5–4.8], respectively, *p* = 0.19) (Figure 2).

**Figure 3:**
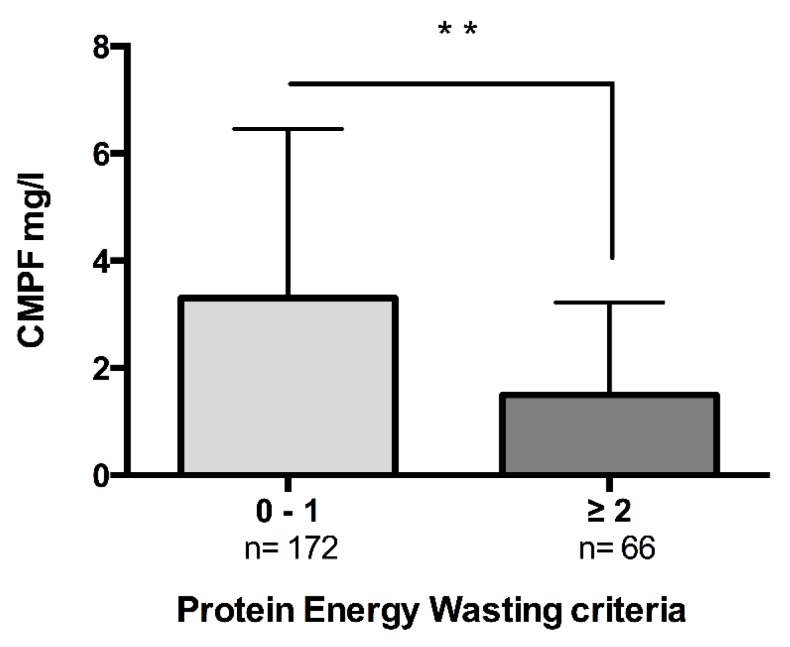
Protein energy wasting is associated with lower serum CMPF levels. Patients were stratified according to the number of PEW criteria. Patients with no or 1 criteria were allocated to the group no PEW (*n* = 172), patients with 2 or more than 2 criteria were allocated to the group PEW (*n* = 66). Data were compared using Mann Whitney U test. Data are expressed as median [IQR]. Differences were considered significant at the *p* < 0.05 level. ** indicates *p* < 0.01. Abbreviation: PEW, protein energy wasting.

We further stratified the patients into two groups according to the number of protein energy wasting (PEW) criteria: there was 172 patients in the group with no PEW and 66 patients in the group with PEW (Table S2). CMPF level was significantly higher in the group without PEW than in the group with PEW (3.3 mg/L [1.2–6.5] versus 1.5 mg/L [0.8–3.2], *p* = 0.0012) (Figure 3). In the whole population CMPF was positively correlated with the lean mass estimated with CI but not with BF (body fat) with anthropometric formula (Table 2). In order to explore if CMPF was associated with body composition, we analysed lean and fat mass with bioimpedancemetry in a subgroup of 66 patients. Characteristics of the subgroup patients are detailed in Table S3, this sub-population did not significantly differ from the whole population (data not shown). Lean body mass, estimated by LTM (lean tissue mass) and BCM (body cell mass), was positively associated with CMPF levels. We did not find any association between CMPF and adiposity estimated either by adipose tissue mass (ATM) or with anthropometric formula (Table 4). BMI, LTI and BCM were not independently associated with CMPF plasma concentration (Table 5).

### 2.3. CMPF Was Not Correlated to Cardiovascular Events and Mortality

**Figure 4:**
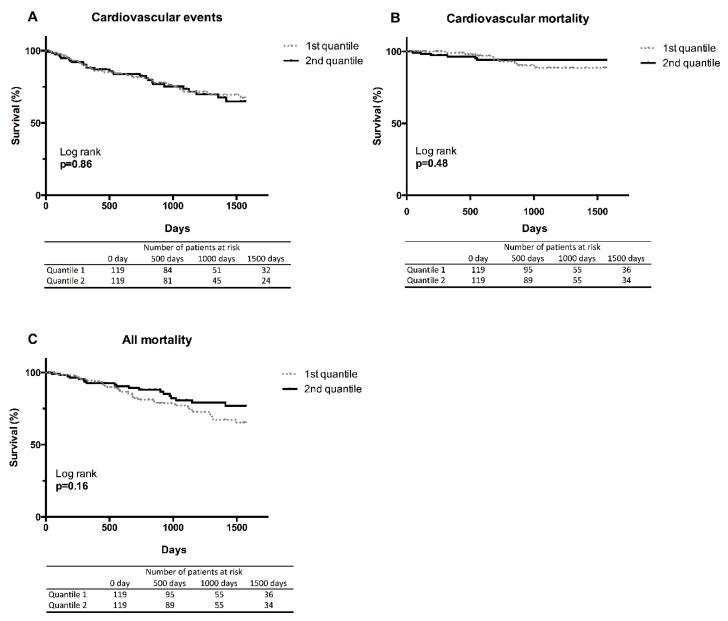
Kaplan–Meier estimates according to serum CMPF concentration at inclusion time. (**A**) Time to first cardiovascular event; (**B**) Cumulative cardiovascular survival; (**C**) Cumulative global survival. Median cut-off (CMPF: quantile 1 < 2.6 mg/L; quantile 2 > 2.6 mg/L. There were 28 cardiovascular events, 9 cardiovascular deaths and a total of 29 deaths in the first quantile. There were 28 cardiovascular events, 6 cardiovascular deaths and a total of 19 deaths in the second quantile.

During the mean period of follow up, 48 patients died, among them, 56 patients underwent a CV events and 15 patients died from a CV events. Kaplan-Meier analysis showed no significant correlation between elevated serum CMPF and an increase of CV event (log rank, *p* = 0.86), CV mortality (log rank, *p* = 0.48) or all-cause mortality (log rank, *p* = 0.16) (Figure 4).

## 3. Discussion

In this large cohort of HD patients, we explored the putative effect of CMPF accumulation on biochemical parameters and mortality and we failed to demonstrate that CMPF is a clinically relevant uremic toxin. We found a positive relationship between CMPF accumulation and nutritional status in HD patients and suggest that CMPF could be an index of a favourable nutritional status. However, this association was not independent after adjustment with albumin, prealbumin, Cr_pre_ and BMI. Then, we found that CMPF was not associated to CV risk and mortality.

Before a retention solute can be accepted as an uremic toxin, EUTox recommended that it should comply with several conditions: (i) the concentration of the compound should be higher in uremic patients than in non-CKD patients; (ii) high concentrations should be related to kidney dysfunction and (iii) biological activity, conforming to clinical changes observed in conjunction with the uremic syndrome, should be proven in in vivo or in vitro studies. Until now CMPF was considered as an uremic toxin [21]. Indeed, numerous studies indicated that CMPF levels were elevated in uraemia [22]. Since the early 1990s, it has been suggested that CMPF could play a role in the pathogenesis of renal dysfunction by activation on the redox system [14]. Several in vitro studies demonstrated ROS toxicity induced by CMPF on tubular cells [14] or on human umbilical vein endothelial cells (HUVEC) [23]. For the first time, in our study, we sought to determine if CMPF accumulation was clinically relevant. Given that, we failed to found any significant association between CMPF and adverse outcomes in HD and we should disqualify this Furan FA from being classified as an uremic toxin but rather as an uremic retention solute (URS).

The conflicting results between clinical and in vitro studies to explore CMPF toxicity could be related to the concentration used for in vitro experiments. Indeed, Table 6 shows the discrepancies between CMPF concentrations reported in publications for HD patients. Fagugli et al. reported the lowest concentration of 3.7 mg/L [24] while the highest concentration reported from Mabuchi H. et al. was 43.9 mg/L in 1987 [25] (Ratio: 11.9). The same discrepancies were observed in healthy population with concentration found to range from 2.3 mg/L to 12 mg/L. Concentrations used for in vitro experiments are consequently variable and therefore difficult to compare. For instance, Prentice et al. have choose a concentration of 48 mg/L for in vitro study on beta-cells whereas Everts et al. choose a concentration of 4.8 mg/L [16]. If a clear toxic effect was observed when cells where treated with high concentration of CMPF (>47 mg/L) [12,14], it was more contradictory with lower concentrations (<10 mg/L) [12,16,23,26]. Moreover, given that CMPF is highly bound to serum albumin, if the quantity of albumin or protein in the in vitro medium are too low or absent, it could give unacceptably high and thus, clinically irrelevant free concentrations [14,23,26]. Surprisingly, in one study, ROS production by CMPF was only observed in the presence of human albumin serum. The authors suggest that this could be due to low solubility of CMPF in water in the absence of human serum albumin [23] (see Table S4). Many factors could account for these differences such as ethnic, genetic background and dietary and/or metabolic factors [22]. Even in the same research unit and using the same methodology, major differences were observed like for the study of Lesaffer et al. [27] (19.7 mg/L) and Fagugli et al. [24] (3.7 mg/L) (ratio: 5.3). Methods used for deproteination, conservation of the samples can also partly explain the variation of CMPF concentrations. Therefore, standardization of extraction methods and measurement is essential to determine the concentration that we should use in vitro to be in realistic concentrations compared with the concentrations observed in human CKD.

CMPF concentration before and after a single HD session has previously been studied. Fagugli et al. showed no differences in plasma CMPF concentration before and after standard or daily HD and even an increase in the concentration due to haemoconcentration [24]. In good agreement, Itoh et al. reported similar results on 45 patients with higher CMPF levels after HD (+30 ± 3%) [23]. The low depuration rate of CMPF during dialysis session is related to its thigh binding to plasma proteins (>95%). This phenomenon could explain the positive correlation we found between CMPF level and dialysis vintage because of accumulation over the years.

Surprisingly, we found that CMPF was significantly correlated with improvement of nutritional status and was significantly lower in patients presenting more than two PEW criteria. Bioimpedancemetry measurement also corroborates the link between CMPF and lean mass. We can hypothesize that CMPF accumulation is an index of a healthier nutritional status. Our results corroborate some observations of a positive relationship between CMPF and healthy diet rich in omega-3 ethyl esters intakes [36]. Unfortunately, in our study nutritional status was only assessed indirectly since no food surveys were performed. This point will deserve further investigations. The association between nutritional parameters and CMPF was lost in multivariate analyses, suggesting that CMPF was not a robust marker.

The main limitation is the absence of nutritional survey for the patients to more accurately explore the nutritional impact of CMPF accumulation and its relationship with omega 3 intakes. Moreover, the population has a globally good nutritional status (see Table 1) and only very few patients exhibited PEW criteria. Another limitation is the absence of information about residual renal function and urine CMPF levels to know if CMPF accumulation is correlated to loss of diuresis. Moreover, CMPF concentration was measured only once at the time of patient inclusion with no further re-evaluation of the plasma level of CMPF. Furthermore, limit of quantification of CMPF concentration with HPLC method was 0.25 mg/L and some patients were below the detection limit. Foremost, the patients studied in this cohort are from the same haemodialysis centre from France and the applicability of the study findings across nationalities remains unclear. Because all our patients were Caucasians our findings could maybe not apply to black patients.

Different studies discussed whether CMPF could be a predictive biomarker for metabolic complications. CMPF exposition in vitro of beta-cells and in vivo of obese or insulin resistant models of mice accelerated diabetes development [37]. CMPF was found to be significantly elevated in the plasma of patients with gestational and type 2 diabetes [29]. Liu Y et al. showed that patient who developed type 2 diabetes had a significant increase in CMPF during the last 4–5 years, while prediabetics patients maintained elevated but stable CMPF levels [37]. In contrast to these metabolic studies, in this prospective cohort of HD patients, we failed to find any association between CMPF levels and metabolic parameters or diabetes. However, this association is not always observed. Savolainen et al. found an inverse correlation between CMPF and the risk of type 2 diabetes development in a population of Swedish women [35] [38]. In good agreement, Retnakaran et al. failed to find any differences in circulating CMPF levels between two groups of women with or without gestational diabetes mellitus [13]. Finally, Lankinen et al. reported that serum CMPF was not associated with impaired glucose tolerance [11]. The reasons for these differences between studies are not clear and need further investigations.

## 4. Conclusions

This is the first large prospective cohort of serum CMPF dosage in HD patients. Our data suggest that CMPF accumulation was not associated to CV risk and mortality in this population. Moreover, in this study, CMPF accumulation was not associated with metabolic disturbances. The deleterious or beneficial effect of CMPF accumulation occurring during CKD remain to be elucidated. This study however brings some evidences to suggest that CMPF is not an uremic toxin and is potentially associated with a better nutritional status, consistent with the hypothesis that CMPF would be a marker of healthy diet and omega 3 intake. Further studies are needed to understand the role of CMPF in HD and to determine its role as a biomarker to assess nutritional status of healthy food dietary in this population.

## 5. Materials and Methods

### 5.1. Ethic Statement

The study protocol was approved by the local ethics committees (DC-2009-1066, CPP Lyon Est IV) and conducted in accordance with its ethical standards and the principles of the second Declaration of Helsinki. All subjects involved in the research signed written informed consent prior to enrolment.

### 5.2. Study Design, Population and Clinical Events

270 prevalent HD patients, older than 18 years old and followed at a single HD centre at AURAL in Lyon, France, were considered for eligibility and invited to participate in this prospective, observational, cohort study. All patients were recruited between 1 March 2012 and 31 December 2015. The inclusion criteria were chronic HD sessions of 4-h, three times every week, for at least 3 months. HD treatments were standardized using high-flux membranes, standard processing techniques and water purification. Exclusion criteria were: current hospitalization, pregnancy, active or invasive malignancy. Patients with local non-melanoma skin cancers, in situ cancer or cancer history (over 5 years old) were included. Demographic factors, relevant medical history and any concomitant medication, were ascertained at the time of the inclusion by review of medical records and patient interviews. For descriptive purposes, patients who reported current or past use of insulin and/or oral hypoglycaemic drugs were considered to have diabetes. Previous CV disease was defined as a history of any of the following events: myocardial infarction, stroke, heart failure, angina pectoris, or surgical procedures for angina or coronary/peripheral artery disease (including percutaneous transluminal angioplasty). Dialysis vintage was defined as the time period between the date of inclusion into the study and the date of the initiation of HD. Dialysis dose was estimated by a spKt/V, as recommended by Daugirdas et al. [39].

During the study period, clinical events, including overall mortality and CV events, were recorded by considering all patients included at least 18 months before the end of the study date (1 July 2016). A physician reviewed each medical chart. For overall mortality and CV events, data were censored at renal transplantation, loss to follow-up, or the end of the study observation period. We defined CV events as CV death or any CV events secondary to CV system dysfunction (stroke, angina pectoris/myocardial infarction, congestive cardiac failure, new-onset arrhythmia or peripheral ischemia) or surgical procedures for angina or coronary/peripheral arterial disease. All deaths attributed to myocardial infarction, cardiogenic shock, peripheral ischemia (including mesenteric ischemia) or stroke were considered as CV deaths. Death occurring outside the hospital for which no other cause was specified was regarded as sudden cardiac death and included in the definition of CV death.

### 5.3. Anthropometric Evaluation and Nutritional Status

Body mass index (BMI) was defined as the post-dialysis dry weight (in kg) divided by the squared height (in m^2^). Body fat mass was calculated using the Deurenberg formula: Body fat = (1.20 × BMI) + (0.23 × Age(years)) − (10.8 × Gender) where male gender = 1, female gender = 0 [40]. Body composition parameters including the lean tissue mass (LTM, kg), lean tissue index (LTI, kg/m^2^) and body cell mass (BCM; the metabolically active component of LTM), adipose tissue mass (ATM, kg) and fat tissue index (FTI, kg/m^2^) were measured in a sub-group of 66 patients, using a bioimpedance spectroscopy (BIS) method (BCM^®^, Fresenius Medical Care, Bad Homburg vor der Höhe, Germany). In order to control for potential variability and the effect of over hydration, all the BIS analyses were performed before a mid-week dialysis session. The BIS monitor uses a whole spectrum of low and high frequencies from 5 to 1000 kHz. BIS–derived body composition estimation was validated against other body composition measures, including magnetic resonance imaging and dual-energy x-ray absorptiometry (DXA) among patients receiving dialysis [41,42,43,44].

Creatinine index, as a surrogate of lean body mass, was calculated from spKt/V, pre-dialysis creatinine serum levels (Crpre) and anthropometric characteristics according to the simplified formula proposed by Canaud et al. [45]: 16.21 + (1.12 × Gender) − (0.06 × Age) − (0.08 × spKt/V) + (0.009 × Crpre(μmol/L), where male gender = 1, female gender = 0.

PEW was assessed if patients presented at least 2 out of 4 criterions: BMI < 23 kg/m^2^, albumin < 38 g/L, pre-albumin < 300 mg/L or normalized protein catabolic rate (nPCR) < 0.8 g/kg per day

### 5.4. Laboratory Measurements

All laboratory data were measured at the baseline visit, in a fasting state, during weekday HD treatments. Serum samples were also collected at the time of the baseline assessment and stored at −20 °C for future use. Biochemical parameters measurements were made using standard methods in the routine clinical laboratory. Albumin was measured by immunonephelometry. Creatinine assay was performed by enzymatic method (Roche, Meylan, France), with calibrators assigned by an isotope-dilution mass spectrometry.

### 5.5. CMPF Assay

CMPF was measured in serum by reverse phase high performance liquid chromatography (See supplementary materials and methods). Briefly, the day of CMPF analysis, serums were left at 4 °C for slow thawing and then vortexed for 15 s. Then, serum was exposed to a heat denaturation of 95 °C for 5 min to separate protein bindings and obtain total CMPF (including both free and bound fraction). Samples were then centrifuged at 13,000 rpm for 60 min at 4 °C. The supernatant was collected and injected in HPLC. HPLC analysis was based on the method published by Mabuchi H. [25]. CMPF was detected at 215 nm. The limit of quantification of CMPF in a sample volume of 20 microliters of human plasma was 0.25 mg/L at a signal to noise ratio of 2:1. The coefficient of variation was 1.4%.

### 5.6. Statistical Analysis

Data were analysed using GraphPad Prism 6.0 (GraphPad software, La Jolla, San Diego, CA, USA) and Statview 5.0 (Abacus concept, Berkeley, CA, USA) softwares. The data are expressed as mean ± SD or as median [IQR] when variable was not normally distributed. Distributions were tested for normality using d’Agostino-Pearson test. Univariate analysis was performed using the Spearman rank correlation method. A multivariate linear regression analysis was used to define the variables most predictive of circulating CMPF concentration after selection of the measures found to be associated with CMPF by univariate analysis (i.e., Crpre, BMI, albumin, prealbumin). HD patients were stratified by increasing concentrations of CMPF into two groups using the median as the cut off: one group low CMPF (*n* = 119) with concentration of less than 2.6 mg/L and high CMPF (*n* = 119) with concentration above 2.6 mg/L for survival analysis. Comparisons between the two groups were assessed with a nonparametric Mann–Whitney test for continuous variables and a chi-squared test for nominal variables. We estimated the association between serum CMF and all-cause mortality, CV mortality and events using Kaplan-Meier plots with the log rank test to compare differences between group. A *p* < 0.05 was considered as statistically significant in all analysis. 

## Figures and Tables

**Table 1:** Clinical and biological characteristics of the haemodialysis population (*n* = 238).

Demographic and Clinical Characteristics		
Age (years)	64.0	[47.2–75.6]
Gender M/F (%)	148/90 (62/38%)	
Dialysis vintage (years)	2.2	[1.1–21.9]
BMI (kg/m^2^)	25.2	[22.6–28.4]
BF (%)	37.8	[31.1–44.3]
CI (mg/kg/day)	19.7	[17.7–21.9]
nPCR	1.1	[0.9–1.3]
spKt/V	1.7	[1.5–1.9]
Medical history of CV events (%)	33%	
HTA (%)	89%	
Type 2 Diabetes (%)	29%	
Type 1 Diabetes (%)	2%	
Dyslipidemia (%)	55%	
Biological characteristics		
Cr_pre_ (μmol/L)	752	[572–1872]
Urea (mmol/L)	20	[16–23]
CMPF (mg/L)	2.55	[1.00–5.23]
Haemoglobin (g/dL)	11.3	[10.5–12.2]
Ferritin (ng/mL)	394	[119–668]
Leukocytes (G/L)	6.1	[5.0–7.3]
Platelets (G/L)	219	[171–268]
Bicarbonate (mmol/L)	21	[19–23]
Phosphorus (mmol/L)	1.4	[1.2–1.8]
Calcaemia (mmol/L)	2.2	[2.1–2.4]
PTH (ng/L)	234	[106–504]
25-OH Vitamin D3 (μg/L)	32	[25–40]
Total cholesterol (g/L)	1.6	[1.4–1.9]
LDL cholesterol (g/L)	0.9	[0.7–1.2]
HDL cholesterol (g/L)	0.4	[0.3–0.5]
Triglycerides (g/L)	1.5	[1.0–2.3]
HbA1c (%)	5.6	[5.6–3.4]
Albumin (g/L)	39.3	[36.3–41.4]
Prealbumin (g/L)	0.33	[0.28–0.39]
CRP (mg/L)	5	[2–12]
NT-proBNP (pg/mL)	1967	[822–4904]

Data are expressed as medians [IQR] or percentages %. Abbreviations: BMI, body mass index, BF, body fat, CI, creatinine index, Cr_pre_, pre-dialysis creatinine serum levels, nPCR, normalized protein catabolic rate, HTA, Hypertension, CMPF, 3-Carboxy-4-methyl-5-propyl-2-furanpropionate, PTH, parathyroid hormone, LDL, low density lipoprotein, HDL, high density lipoprotein, HbA1c, glycated haemoglobin, CRP, c-protein reactive, NT-proBNP, N- terminal pro-brain natriuretic peptide, spKt/V, single-pool Kt/V.

**Table 2:** Unadjusted Spearman Correlation Coefficients (r_s_) of CMPF and Other Relevant Covariates in Haemodialysis Patients.

Variable	r_s_	95%CI	*p*-Value
Age (years)	−0.07	[−0.20 to 0.06]	0.28
Dialysis vintage (years)	0.16	[0.03 to 0.29]	**0.01**
spKt/V	−0.09	[0.22 to 0.04]	0.18
BMI (kg/m^2^)	0.16	[0.03 to 0.30]	**0.01**
BF (%)	0.11	[−0.02 to 0.24]	0.10
CI (mg/kg/day)	0.16	[0.03 to 0.29]	**0.01**
Haemoglobin (g/dL)	0.24	[−0.06 to 0.21]	0.24
Leukocytes (/mm^3^)	−0.13	[−0.26 to −0.00]	**0.04**
Platelets (/mm^3^)	−0.10	[−0.23 to 0.03]	0.12
Cr_pre_ (μmol/L)	0.18	[0.05 to 0.31]	**0.004**
Urea (mmol/L)	0.09	[−0.04 to 0.22]	0.18
Bicarbonate (mmol/L)	0.05	[−0.08 to 0.18]	0.41
Calcaemia (mmol/L)	−0.01	[−0.14 to 0.12]	0.91
Phosphoremia	−0.03	[−0.16 to 0.10]	0.65
Parathormon (ng/L)	0.12	[−0.02 to 0.25]	0.09
25-OH Vitamin D3 (μg/L)	0.12	[−0.02 to 0.25]	0.12
CRP (mg/L)	0.02	[−0.11 to 0.16]	0.74
Glycaemia (g/L)	−0.08	[−0.21 to 0.05]	0.22
HbA1c (%)	−0.01	[−0.17 to 0.15]	0.87
Total cholesterol (g/L)	−0.01	[−0.17 to 0.14]	0.88
LDL cholesterol (g/L)	−0.08	[−0.24 to 0.08]	0.31
HDL cholesterol (g/L)	−0.09	[−0.24 to 0.07]	0.25
Triglycerides (g/L)	0.08	[−0.08 to 0.23]	0.33
Albumin (g/L)	0.20	[0.07 to 0.32]	**0.003**
Pre-albumin (g/L)	0.16	[0.02 to 0.29]	**0.02**
nPCR (Garred)	−0.01	[−0.15 to 0.13]	0.89
NT-proBNP (pg/mL)	−0.14	[−0.27 to −0.00]	**0.04**

Abbreviations: BMI, body mass index, BF, body fat, CI, creatinine index, Cr_pre_: pre-dialysis creatinine nPCR, normalized protein catabolic rate, HTA, Hypertension, CMPF, 3-Carboxy-4-methyl-5-propyl-2-furanpropionate, PTH, parathyroid hormone, LDL, low density lipoprotein, HDL, high density lipoprotein, HbA1c, glycated haemoglobin, CRP, c-protein reactive, NT-proBNP, N-terminal pro-brain natriuretic peptide, spKt/V: single-pool Kt/V.

**Table 3:** Multiple linear regression of association with CMPF concentration.

Dependent Variable: CMPF			
Independent Variable	β Coefficient	Standard Error	*p*-Value
Albumin (g/L)	0.232	0.191	0.23
Prealbumin (g/L)	0.003	0.008	0.73
Cr_pre_ (μmol/L)	0.003	0.003	0.33
BMI (kg/m^2^)	0.062	0.103	0.55
Intercept	−8.442	7.163	

Abbreviations: BMI, body mass index, Cr_pre_, pre-dialysis creatinine, CMPF, 3-Carboxy-4-methyl-5-propyl-2-furanpropionate.

**Table 4:** Unadjusted Spearman Correlation Coefficients (r_s_) of CMPF and bioimpedance parameters.

Variable	r_s_	95%CI	*p*-Value
BMI (kg/m^2^)	0.43	[0.20 to 0.62]	<0.001
Lean tissue mass (LTM) (kg)	0.29	[0.046 to 0.50]	0.02
Lean tissue index (LTI) (kg/m^2^)	0.36	[0.12 to 0.56]	0.003
Adipose tissue mass (ATM) (kg)	0.09	[−0.33 to 0.17]	0.49
Fat tissue index (FTI) (kg/m^2^)	0.10	[−0.34 to 0.16]	0.45
Body cell mass (BCM) (kg)	0.31	[0.07 to 0.52]	0.01

Correlation spearman test was performed in a subgroup of 66 haemodialysis patients. Abbreviations: BMI, body mass index, LTM, lean tissue mass, LTI, lean tissue index, ATM, adipose tissue mass, FTI, fat tissue index, BCM, body cell mass.

**Table 5:** Multiple linear regression of association and lean body mass with CMPF concentration.

Dependent Variable: CMPF			
Independent Variable	β Coefficient	Standard Error	*p*-Value
BMI (kg/m^2^)	0.025	3.206E^−4^	0.85
Lean tissue index (LTI) (kg/m^2^)	0.525	5.397	0.38
Body cell mass (BCM) (kg)	−0.320	2.528	0.59
Intercept	−9.394		

Multiple linear regression test was performed in a subgroup of 66 haemodialysis patients. Abbreviations: BMI, body mass index, LTI, lean tissue index, BCM, body cell mass.

**Table 6:** CMPF concentration reported in populations of haemodialysis patients.

	Study	HD Technique	PreHD Total CMPF (mg/L)		Patients	Detection Technique
1987	**Mabuchi H.** [25]	HD	43.9	±9.1	*n* = 13	HPLC—UV detection 215 nm
1990	**Niwa T.** [28]	HD	41.0	±18.3	*n* = 23	HPLC—UV detection 270 nm
1994	**Niwa T.** [29]	HD	32.3	±13.2	*n* = 20	HPLC—UV detection 270 nm
2000	**Lesaffer G.** [27]	High Flux polysulphone HD	19.7	±10.3	*n* = 10	HPLC—UV detection 254 nm
		High Flux cellulose triacetate HD	17.6	±7.7	*n* = 10	
		Low flux polysulphone HD	17.1	±8.9	*n* = 10	
2000	**Sassa T.** [30]	HD	32.3	±2.7	*n* = 17	HPLC—UV detection 270 nm
2002	**Fagugli M. R.** [24]	Standard HD (SHD)	3.7	±2.5	*n* = 7	HPLC—UV detection 254 nm
		Daily HD (DHD)	3.6	±2.0	*n* = 7	
2007	**De Smet R.** [31]	Low Flux HD	8.8	±5.0	*n* = 11	HPLC—UV detection 254 nm
		Super Flux cellulose triacetate HD	8.4	±3.6		
2008	**Nishio T.** [32]	HD	18.8	±5.8	*n* = 14	HPLC—UV detection 261 nm
2010	**Brandenburg V.** [33]	HD	4.0	±2.9	*n* = 41	HPLC—UV detection 254 nm
2012	**Itoh Y.** [23]	HD	21.1	±1.3	*n* = 45	LC/ESI-MS/MS
2013	**Eloot S.** [34]	HD	3.8 [2.0–6.1]		*n* = 71	HPLC—UV detection 254 nm
2016	**Rroji M.** [35]	HD	4.3 [2.3–7.7]		*n* = 126	RP-HPLC—UVdetection254 nm
2018	**Luce M.**	HD	2.5 [1.0–5.2]		*n* = 238	HPLC—UV detection 215 nm

Data are presented as mean ± SD or median [interquartile range]. Abbreviations: HD, haemodialysis, HPLC, high-performance liquid chromatography, LC/ESI-MS/MS, liquid chromatography/electrospray ionization–mass spectrometry/mass spectrometry.

## Supplementary Materials

The following are available online at http://www.mdpi.com/2072-6651/10/5/205/s1, Table S1. Baseline characteristics of the patient with low and high CMPF. Table S2. Baseline characteristics of the patient without and with PEW criteria. Table S3. Characteristics of patients with bioimpedance measurement. Table S4: Effect of CMPF in different cellular models.

## Appendix A. Supplementary Material and Methods

### CMPF Assay

#### Appendix A.1.1. Stock Solutions, Calibration Standards and Quality-Control (QC) Samples of CMPF

A stock solution (5 mg/mL) of CMPF (Cayman chemicals, Ann Arbor, MI, USA) was prepared in DMSO, aliquoted and stored at −20 °C. A standard solution containing 250 mg/L (1040 µM) of CMPF was prepared from the stock solution by serial dilution in methanol. Calibration standards were prepared at 250, 125, 62.5, 31.3, 15.6, 7.8, 3.9 and 2 mg/L and subjected to HPLC analysis. Quality control (QC) samples were prepared from low to high concentrations of CMPF found during CKD in the literature. CMPF from stock solution was added to healthy volunteers’ sera for final CMPF concentration of 25, 50, 75, 100 and 125 mg/L. QC samples were left one hour at 4 °C to allow protein bindings before conditioning for HPLC analysis. All calibration standards and QC samples were prepared on the day of the analysis.

#### Appendix A.1.2. Serum Sample Preparation

Venous blood samples were centrifugated immediately after collection (15 min, 3500 g). The supernatant serum was collected and frozen for storage at −20 °C. The day of CMPF analysis, serum was left at 4 °C for slow thawing and then vortexed for 15 s. 350 µL of serum was exposed to a heat denaturation of 95 °C for 5 min to denaturate proteins and obtain total CMPF (including both free and bound fraction). Samples were then centrifuged at 13,000 rpm for 60 min at 4 °C. The supernatant was collected and 20 µL was injected in HPLC.

#### Appendix A.1.3. Chromatography

HPLC analysis was based on the method published by Mabuchi H. [25] with modifications. The HPLC system consisted of an Agilent technologies LC 1200 series system coupled to an UV-visible diode array detector. A RP-C18 Spherisorb ODS2 column (5 µm, 4.6 × 250 mm, Grace Discovery Sciences, Epernon, France) was used as the stationary phase. The mobile phase consisted of 40% (*v*/*v*) acetonitrile 60% (*v*/*v*) ultrapure water acidified with 0.2% (*v*/*v*) of acetic acid. CMPF was detected at 215 nm. The mobile phase flow was 1 ml/min during the procedure and total run time was 7 min for each sample. Twenty microliters of calibration standards or deproteinized serum were automatically injected on the HPLC system.

#### Appendix A.1.4. Validation of HPLC Technique

The assay was validated for selectivity, calibration range, accuracy and precision. Nature of the compound and therefore selectivity was assessed for the specific UV spectrum of CMPF peaks comparing to previous studies and to standard compounds. A range of calibration standards with 8 points calibration was prepared 5 times and injected in HPLC to determine the calibration curve standard, the concentrations used are detailed above. Calibration standards were analysed by HPLC before and after heat shock to attest CMPF thermo-stability. To define the accuracy and precision of the method, we prepared samples of serum from HV with a specific concentration of CMPF described in the section above. Accuracy was defined as the difference between calculated and the exact amount of CMPF and was expressed as a percentage. Precision was assessed as percentage relative standard deviation (% RSD) for the related CMPF concentration. Limit of detection was determined according to the protocol of the Environmental Protection Agency (EPA)*.*

The standard curve of CMPF passed through the origin and was linear up to a concentration of 250 mg/L. The limit of quantification of CMPF in a sample volume of 20 µL of human plasma was 0.25 mg/L at a signal to noise ratio of 2:1. The coefficient of variation was 1.4%.
