# Supplementary material for: Is 3-Carboxy-4-methyl-5-propyl-2-furanpropionate (CMPF) a Clinically Relevant Uremic Toxin in Haemodialysis Patients?
Source: Toxins (Basel). 2018 May 18;10(5):205. doi: 10.3390/toxins10050205 (PMC5983261; doi:10.3390/toxins10050205)
Supplement: Supplementary file 1 [file toxins-10-00205-s001.pdf]

Supplementary Materials: Is 3-Carboxy-4-methyl-5-propyl-2-furanpropionate (CMPF) a Clinically Relevant Uremic Toxin in Haemodialysis Patients?

Mathilde Luce, Anais Bouchara, Myriam Pastural, Samuel Granjon, Jean Christophe Szelag, Maurice Laville, Walid Arkouch, Denis Fouque, Christophe O. Soulage and Laetitia Koppe

Table S1. Baseline characteristics of the patient with low and high CMPF.

	**CMPF < 2.6 mg/l (n=119)**		**CMPF ≥ 2.6 mg/l (n=119)**		**p-value**
**Clinical characteristics**					
Age (years)	65.9	[46.4 - 77.3]	61.5	[51.1 - 73.5]	0.39
Sex male/female	73/46		75/44		0.78
Dialysis vintage (years)	1.8	[1.0 - 4.2]	2.5	[1.1 - 5.3]	**0.08**
BMI (kg/m^2)	24.5	[20.7 - 27.7]	25.8	[23.4 - 29.7]	**0.009**
CI (kg/mg/day)	19.5	[17.2 - 21.5]	20.1	[18.2 - 22.4]	**0.02**
nPCR	1.1	[0.9 - 1.2]	1.1	[0.9 - 1.3]	0.42
spKt/V	1.7	[1.5 - 1.9]	1.6	[1.4 - 1.8]	0.57
Medical history of CV events (%)	31		35		0.49
HTA (%)	89		89		1
Type 1 or 2 Diabetes (%)	34		26		0.20
Dyslipidemia (%)	54		56		0.70
**Biological characteristics**					
Cr_{pre} (µmol/l)	698	[546 - 864]	796	[628 - 940]	**0.004**
Urea (mmol/l)	19	[16 - 22]	21	[18 - 24]	**0.04**
Hemoglobin (g/dl)	11.3	[10.4 - 12]	11.3	[10.5 - 12.3]	0.55
Ferritin (ng/ml)	368	[176 - 735]	400	[209 - 628]	0.89
Leukocytes (G/l)	6.3	[5.1 - 7.5]	5.8	[4.8 - 7.1]	**0.07**
Platelets (G/l)	226	[182 - 282]	213	[165 - 257]	0.12
Bicarbonate (mmol/l)	21	[19 - 23]	21	[19 - 23]	0.82
Phosphorus (mmol/l)	1.4	[1.1 - 1.8]	1.5	[1.2 - 1.8]	0.93
Calcemia (mmol/l)	2.2	[2.1 - 2.3]	2.6	[2.1 - 2.4]	0.87
PTH (ng/l)	217	[97 - 472]	272	[112 - 514]	0.35
25-OH Vitamin D3 (µg/l)	31	[23 - 37]	35	[25 - 42]	**0.05**
Total cholesterol (g/l)	1.7	[1.4 - 2.0]	1.6	[1.3 - 1.9]	0.31
LDL cholesterol (g/l)	0.93	[0.78 - 1.26]	0.86	[0.61 - 1.10]	**0.04**
HDL cholesterol (g/l)	0.39	[0.30 - 0.49]	0.38	[0.30 - 0.38]	0.27
Triglycerides (g/l)	1.5	[1.0 - 2.1]	1.7	[1.1 - 2.4]	0.26
HbA1c (%)	5.6	[5.0 - 6.6]	5.6	[5.1 - 6.1]	0.80
Albumin (g/l)	38.5	[35.7 - 40.6]	40.0	[37.2 - 41.6]	**0.007**
Prealbumin (g/l)	0.32	[0.27 - 0.38]	0.35	[0.29 - 0.30]	**0.02**
CRP (mg/l)	4.9	[2.0 - 11.2]	5.7	[1.9 - 13.7]	0.47
NT-proBNP (pg/ml)	2590	[910 - 5762]	1619	[725 - 4379]	0.10

Data are expressed as medians [IQR] or percentages (%).
Abbreviations: BMI, body mass index, CI, creatinine index, nPCR, normalized protein catabolic rate, Crpre: pre-dialysis creatinine, HTA, Hypertension, CMPF, 3-Carboxy-4-methyl-5-propyl-2-furanpropionate, PTH, parathyroid hormone, LDL, low density lipoprotein, HDL, high density lipoprotein, HbA1c, glycated haemoglobin, CRP, c-protein reactive, NT-proBNP, N- terminal pro-brain natriuretic peptide, sp Kt/V: single-pool Kt/V

Table S2. Baseline characteristics of the patients without and with PEW criteria.

	**0-1 PEW criteria (n=172)**		**≥ 2 PEW criteria (n=66)**		**p-value**
**Clinical characteristics**					
Age (years)	59.2	[44.9 - 72.3]	71.6	[51.8 - 79.3]	**0.009**
Sex male/female	109/63		39/27		0.54
Dialysis vintage (years)	2.3	[1.1 -4.7]	2.0	[1.1 - 4.2]	0.83
BMI (kg/m²)	25.7	[23.4 - 29.4]	23.7	[20.4 - 27.1]	**0.003**
CI (kg/mg/day)	20.3	[18.3 - 22.4]	17.9	[16.9 - 20.3]	**<0.0001**
nPCR	1.10	[0.96 - 1.30]	0.99	[0.77 - 1.24]	**0.003**
spKt/V	1.6	[1.4 - 1.8]	1.8	[1.5 - 2.0]	**0.009**
Medical history of CV events (%)	31		38		0.95
HTA (%)	87		94		0.14
Type 1 or 2 Diabetes (%)	29		32		0.68
Dyslipidemia (%)	55		55		0.92
**Biological characteristics**					
Cr_{pre} (µmol/l)	796	[620 - 937]	622	[520 - 788]	**<0.0001**
Urea (mmol/l)	20.8	[18.0 - 23.6]	17.6	[13.3 - 21.6]	**<0.0001**
CMPF (mg/l)	3.3	[1.2 - 6.5]	1.5	[0.8 - 3.2]	**0.001**
Hemoglobin (g/dl)	11.3	[10.5 - 12.2]	11.1	[10.3 - 12.2]	0.46
Ferritin (ng/ml)	407	[194 - 680]	342	[141 - 655]	0.57
Leukocytes (G/l)	5.9	[5.1 - 7.1]	6.3	[4.9 - 7.8]	0.21
Platelets (G/l)	218	[169 - 258]	227	[182 - 285]	0.17
Bicarbonate (mmol/l)	21	[19 - 23]	22	[20 - 25]	**0.01**
Phosphorus (mmol/l)	1.5	[1.2 - 1.8]	1.2	[1.0 - 1.7]	**0.01**
Calcemia (mmol/l)	2.3	[2.1 - 2.4]	2.2	[2.1 - 2.3]	0.12
PTH (ng/l)	260	[120 - 514]	182	[67 - 468]	**0.03**
25-OH Vitamin D3 (µg/l)	34	[26 - 41]	30	[20.3 - 35.8]	**0.02**
Total cholesterol (g/l)	1.6	[1.4 - 1.9]	1.7	[1.3 - 2.0]	0.57
LDL cholesterol (g/l)	0.9	[0.7 - 1.1]	0.9	[0.7 - 1.3]	0.32
HDL cholesterol (g/l)	0.4	[0.3 - 0.5]	0.4	[0.3 - 0.5]	0.97
Triglycerides (g/l)	1.6	[1.0 - 2.3]	1.5	[1.1 - 2.1]	0.58
HbA1C (%)	5.5	[5.0 - 6.1]	5.7	[5.1 - 6.7]	0.31
Albumin (g/l)	40.2	[38.2 - 41.9]	35.6	[25.5 - 37.0]	**<0.0001**
Prealbumin (g/l)	0.36	[0.32 - 0.41]	0.27	[0.23 - 0.29]	**<0.0001**
CRP (mg/l)	4.1	[1.7 - 9,1]	10.5	[3.4 - 19.7]	**<0.0001**
NT-proBNP (pg/ml)	1689	[730 - 4049]	2864	[1215 - 6273]	**0.009**

Data are expressed as medians [IQR] or percentages (%).
Abbreviations: BMI, body mass index, CI, creatinine index, nPCR, normalized protein catabolic rate, Crpre: pre-dialysis creatinine, HTA, Hypertension, CMPF, 3-Carboxy-4-methyl-5-propyl-2-furanpropionate, PTH, parathyroid hormone, LDL, low density lipoprotein, HDL, high density lipoprotein, HbA1c, glycated haemoglobin, CRP, c-protein reactive, NT-proBNP, N- terminal pro-brain natriuretic peptide, spKt/V: single-pool Kt/V

Table S3. Characteristics of patients with bioimpedance measurement (n=66).

**Clinical characteristics**		
Age (years)	6.33	[47.2 - 74.3]
Male (%)	41	
Women (%)	59	
Dialysis vintage (years)	2.8	[1.1 - 5.0]
BMI (kg/m^2)	25.2	[22.2 - 28.0]
Creatine Index (kg/mg/day)	19.9	[17.5 - 19.9]
BF (%)	38.2	[31.6 - 44.0]
Lean tissue mass (LTM) (kg)	36.9	[28.4 - 43.6]
Lean tissue index (LTI) (kg/m^2)	12.7	[10.6 - 15.4]
Adipose tissue mass (ATM) (kg)	33.9	[20.6 - 47.0]
Fat tissue index (FTI) (kg/m^2)	12.9	[7.6 - 17.8]
Body cell mass (BCM) (kg)	19.0	[14.5 - 25.3]
nPCR	1.07	[0.93 - 1.30]
sp Kt/V	1.6	[1.5 - 1.8]
Medical history of CV events (%)	30	
HTA (%)	88	
Type 2 diabetes (%)	27	
Type 1 diabetes 1	3	
Dyslipidemia (%)	58	
**Biological characteristics**		
Cr_{pre} (µmol/l)	787	[584 - 943]
Urea (mmol/l)	20.1	[17.5 - 22.2]
CMPF (mg/l)	3.30	[1.40 - 6.93]
Hemoglobin (g/dl)	11.2	[10.7 - 12.1]
Ferritin (ng/ml)	437	[215 - 775]
Leukocytes (G/l)	5.9	[5.0 - 7.0]
Platelets (G/l)	218	[175 - 262]
Bicarbonate (mmol/l)	21	[19 - 23]
Phosphorus (mmol/l)	1.5	[1.2 - 1.8]
Calcemia (mmol/l)	2.2	[2.1 - 2.4]
PTH (ng/l)	367	[192 - 588]
25-OH Vitamin D3 (µg/l)	31	[21 - 38]
Total cholesterol (g/l)	1.70	[1.39 - 2.16]
LDL cholesterol (g/l)	0.86	[0.70 - 1.26]
HDL cholesterol (g/l)	0.35	[0.3 - 0.49]
Triglycerides (g/l)	1.60	[1.04 -2.20]

HbA1c (%)	5.4	[5.0 - 6.0]
Albumin (g/l)	39.5	[36.4 - 42.1]
Prealbumin (g/l)	0.34	[0.29 - 0.40]
CRP (mg/l)	4.7	[2.1 - 11.4]
NT-proBNP (pg/ml)	1379	[662 - 2840]

Data are expressed as medians [IQR] or percentages %.
Abbreviations: BMI, body mass index, BF, body fat percentage, nPCR, normalized protein catabolic rate, Crpre: pre-dialysis creatinine, HTA, Hypertension, CMPF, 3-Carboxy-4-methyl-5-propyl-2-furanpropionate, PTH, parathyroid hormone, LDL, low density lipoprotein, HDL, high density lipoprotein, HbA1c, glycated haemoglobin, CRP, c-protein reactive, NT-proBNP, N- terminal pro-brain natriuretic peptide, sp Kt/V: single pool Kt/V

Table S4. Effect of CMPF in different cellular models.

References	Type of cells used for in vitro experiments	CMPF actions	CMPF concentration
Niwa et al [1]	Isolated mitochondria	Inhibition of ADP-stimulated oxidation of NADH-linked substrates.	48 mg/l (200 μM)
Lim et al. [2]	Rats hepatocytes	Inhibition of iodide production (deiodination of T4).	2,4 mg/l (10 μM)
Everts et al. [3]	Anterior pituitary cells	Increased concentrations of T4.<br>Reduced T4 uptake.<br>No effect on TSH released.	4,8 mg/l (20 μM)<br>48 mg/l (200 μM)
Sun et al. [4]	Rats hepatocytes	CMPF reduced hepatocyte uptake of erythromycin<br>Inhibition of the enzymatic metabolism of erythromycin by demethylation.	6 mg/l (25 μM)<br>48 mg/l (200 μM)
Volpe et al. [5]	Human liver microsomes	Effect of CMPF on drug metabolism: CMPF inhibited CYP3A4 metabolism (testosterone metabolism).	20,7 mg/l (86 μM) (IC50 value)
Prentice et al. [6]	β cells and mice islets	CMPF impairs glucose tolerance and utilization.<br>Reduction in high glucose-stimulated secretion.	36 mg/l (150 μM) and 48 mg/l (200 μM)
Miyamoto et al [7]	Proximal tubular HK-2 cells	CMPF increases active TGF-β1secretion in the presence of Angiotensin II and iron.<br>CMPF in addition of Angiotensin II and iron resulted in a significant increase in ROS production.<br>CMPF directly interacts with superoxide anion radicals and peroxy-radicals to produce CMPF radicals.<br>CMPF itself did not increase intracellular ROS production.	96 mg/l (400 μM)
Itoh et al. [8]	HUVEC	Increase in ROS production in the presence of 4 % HSA.<br>No significant increased ROS production at 21 mg/l (87μM) or without HSA.	47 mg/l (196 μM)
Tsujimoto et al. [9]	Caco-2 cells<br>Hep3B cells	Expression level of MRP2 mRNA tended to decrease.<br>Expression level of OATP1B1 mRNA in Hep3B cells tended to decrease.	9.6 mg/l (40 μM)

Abbreviations: HUVEC, human umbilical vein endothelial cells, NADH, nicotinamide adenine dinucleotide, T4, thyroxin, TGF-β1, Transforming growth factor beta 1, ROS, reactive oxygene species, HSA, human serum albumin.

References

1. Niwa, T.; Aiuchi, T.; Nakaya, K.; Emoto, Y.; Miyazaki, T.; Maeda, K. Inhibition of mitochondrial respiration by furancarboxylic acid accumulated in uremic serum in its albumin-bound and non-dialyzable form. *Clin. Nephrol.* **1993**, *39*, 92–96.
2. Lim, C.F.; Bernard, B.F.; de Jong, M.; Docter, R.; Krenning, E.P.; Hennemann, G. A furan fatty acid and indoxyl sulfate are the putative inhibitors of thyroxine hepatocyte transport in uremia. *J. Clin. Endocrinol. Metab.* **1993**, *76*, 318–324, doi:10.1210/jcem.76.2.8432774.
3. Everts, M.E.; Lim, C.F.; Moerings, E.P.; Docter, R.; Visser, T.J.; De Jong, M.; Krenning, E.P.; Hennemann, G. Effects of a furan fatty acid and indoxyl sulfate on thyroid hormone uptake in cultured anterior pituitary cells. *Am. J. Physiol.* **1995**, *268*, E974–E979, doi:10.1152/ajpendo.1995.268.5.E974.
4. Sun, H.; Huang, Y.; Frassetto, L.; Benet, L.Z. Effects of uremic toxins on hepatic uptake and metabolism of erythromycin. *Drug Metab. Dispos. Biol. Fate Chem.* **2004**, *32*, 1239–1246, doi:10.1124/dmd.104.000521.
5. Volpe, D.A.; Tobin, G.A.; Tavakkoli, F.; Dowling, T.C.; Light, P.D.; Parker, R.J. Effect of uremic serum and uremic toxins on drug metabolism in human microsomes. *Regul. Toxicol. Pharmacol. RTP* **2014**, *68*, 297–303, doi:10.1016/j.yrtph.2013.10.006.
6. Prentice, K.J.; Luu, L.; Allister, E.M.; Liu, Y.; Jun, L.S.; Sloop, K.W.; Hardy, A.B.; Wei, L.; Jia, W.; Fantus, I.G.; et al. The Furan Fatty Acid Metabolite CMPF Is Elevated in Diabetes and Induces β Cell Dysfunction. *Cell Metab.* **2014**, *19*, 653–666, doi:10.1016/j.cmet.2014.03.008.
7. Miyamoto, Y.; Iwao, Y.; Mera, K.; Watanabe, H.; Kadowaki, D.; Ishima, Y.; Chuang, V.T.G.; Sato, K.; Otagiri, M.; Maruyama, T. A uremic toxin, 3-carboxy-4-methyl-5-propyl-2-furanpropionate induces cell damage to proximal tubular cells via the generation of a radical intermediate. *Biochem. Pharmacol.* **2012**, *84*, 1207–1214, doi:10.1016/j.bcp.2012.07.033.
8. Itoh, Y.; Ezawa, A.; Kikuchi, K.; Tsuruta, Y.; Niwa, T. Protein-bound uremic toxins in hemodialysis patients measured by liquid chromatography/tandem mass spectrometry and their effects on endothelial ROS production. *Anal. Bioanal. Chem.* **2012**, *403*, 1841–1850, doi:10.1007/s00216-012-5929-3.
9. Tsujimoto, M.; Hatozaki, D.; Shima, D.; Yokota, H.; Furukubo, T.; Izumi, S.; Yamakawa, T.; Minegaki, T.; Nishiguchi, K. Influence of serum in hemodialysis patients on the expression of intestinal and hepatic transporters for the excretion of pravastatin. *Ther. Apher. Dial.* **2012**, *16*, 580–587, doi:10.1111/j.1744-9987.2012.01100.x.
